# Exploring Patterns of Medication Usage in Affective Disorder Patients: A Comprehensive Investigation at a Psychiatry Outpatient Clinic of a Tertiary Care Hospital

**DOI:** 10.7759/cureus.60290

**Published:** 2024-05-14

**Authors:** Urvashi S Modh, Satish Suthar, Priyadarshini M Zula, Monika Patel, Anurag K Pipalava, Rohankumar Gandhi

**Affiliations:** 1 Pharmacology, GMERS (Gujarat Medical Education and Research Society) Medical College, Junagadh, IND; 2 Pharmacology, Shri M. P. Shah Government Medical College, Jamnagar, IND; 3 Clinical Pharmacology, Postgraduate Institute of Medical Education and Research, Chandigarh, IND; 4 Community and Family Medicine, Shri M. P. Shah Government Medical College, Jamnagar, IND

**Keywords:** prescribed daily dose (pdd), drug utilization, prescription patterns, defined daily dose (ddd), rational drug use

## Abstract

Introduction: Affective disorders impose a significant burden on public health due to their high prevalence and associated suffering. This study addresses gaps in current literature and clinical practice by providing insights into medication usage trends, which can inform treatment strategies and optimize patient care. The study aims to investigate drug utilization patterns, particularly focusing on defined daily dose/1000/day, among individuals attending a psychiatric outpatient department of a tertiary care hospital.

Methods: This cross-sectional, prospective drug utilization study included 600 affective disorder patients aged 18 years and above. The study period spanned 12 months, from March 2021 to February 2022. Data on demographics, diagnosis, treatment, and counseling were collected and analyzed using descriptive statistics.

Results: Among the 600 patients analyzed, bipolar mood disorder was the most prevalent (239 patients, 39.83%), followed by depressive disorder (208 patients, 34.67%). Triple therapy was the most common prescription regimen, accounting for 308 encounters (51.33%). The average number of drugs per encounter was 3.75 ± 1.01. A combination of psychotherapy and medication counseling sessions was provided to 594 patients or their relatives, representing 99% of the total encounters.

Conclusion: The study highlights the prevalent use of triple therapy in managing affective disorders, especially bipolar mood disorder and mania disorder. Effective utilization of essential drug lists and comprehensive patient counseling underscores the importance of holistic care in psychiatric outpatient settings.

Recommendation: Given the high prevalence of triple therapy, further research into the efficacy and safety of this treatment approach is warranted. Additionally, continued emphasis on patient education and counseling can enhance treatment adherence and overall outcomes in individuals with affective disorders.

## Introduction

Affective disorders, characterized by a high prevalence, inflict substantial suffering, dysfunction, and economic burden, making them a matter of major public health concern. The prolonged treatment of affective disorder patients with antidepressants, mood stabilizers, and other medications imposes a considerable strain on healthcare resources [1]. Recognizing this substantial burden, our study aims to conduct a drug utilization investigation, focusing on key indicators such as defined daily dose (DDD)/1000/day, among individuals attending the psychiatric outpatient department of a government tertiary care hospital.

Pharmacoepidemiology, situated at the intersection of pharmacology and epidemiology, serves as a crucial discipline for studying the interaction between drugs and populations. Its primary objectives involve evaluating the utilization and impact of healthcare products within the actual treated population, transcending theoretical target populations defined in pre-marketing trials and marketing authorizations [2]. Within pharmacoepidemiology, drug utilization research stands out as an essential component, detailing the extent, nature, and determinants of drug exposures [3]. These studies play a pivotal role as powerful exploratory tools, shedding light on the societal role of drugs and forming the socio-medical and health economic foundation for healthcare decision-making [4]. In our study, we will utilize pharmacoepidemiological methods to examine the drug utilization patterns and health outcomes within affective disorder patients. This approach will clarify the relevance of pharmacoepidemiology to our research objectives and enhance the coherence of our study design.

According to the World Health Organization (WHO), drug utilization research encompasses the marketing, distribution, prescription, and usage of drugs in society, with a special focus on the resulting medical, social, and economic consequences [5]. The overarching goal of drug utilization research is to facilitate the rational use of drugs in populations. With a proliferation of pharmaceutical products globally and a subsequent surge in drug consumption and expenditures, it becomes imperative to examine the patterns of drug utilization, particularly in regions where accessibility remains a challenge [6].

Physician prescribing behavior is influenced by diverse sources such as patient input, commercial promotions, professional advice, academic literature, and government regulations. Notably, irrational drug prescriptions are prevalent, often attributed to a lack of drug knowledge, unethical promotion, and irrational prescribing habits among clinicians [7]. Our study seeks to address these issues through the monitoring of prescriptions and drug utilization studies, providing valuable feedback to prescribers. In resource-constrained developing countries, where healthcare funds are limited, the need for rational drug prescribing becomes paramount to ensure optimal utilization of available resources [8]. Affective disorders present specific challenges and notable patterns in drug utilization that differentiate them from other medical conditions. For instance, long-term medication adherence is often a significant issue due to the chronic nature of affective disorders. Additionally, the complexity of managing comorbid conditions, such as substance abuse or other psychiatric disorders, adds another layer of complexity to treatment.

The concept of essential drugs further underlines the importance of drug availability for meeting the healthcare needs of the majority. These drugs should be consistently accessible in adequate quantities, in appropriate dosage forms, and at affordable prices, aligning with national responsibilities [9]. There is a dearth of studies examining drug utilization patterns in affective disorders within this geographical area. Therefore, our study aims to unravel the complex landscape of drug utilization in affective disorder patients, providing insights to enhance rational drug use and optimize healthcare resources.

## Materials and methods

This cross-sectional, prospective drug utilization study was conducted in the psychiatric outpatient department of a tertiary care hospital (Guru Gobind Singh Government Hospital). The study period spanned 12 months, from March 2021 to February 2022. Prior approvals were obtained from the medical superintendent, Institutional Ethical Committee, and the Head of the Psychiatry Department.

Patient selection criteria

Inclusion criteria were patients aged 18 years and above of any gender attending the psychiatric outpatient department (OPD). The diagnosis was established based on clinical assessment by the on-duty psychiatrist at the hospital.

Patients meeting any of these criteria were excluded from the study: individuals with uncertain diagnoses, pediatric patients under the age of 18 years, pregnant and lactating mothers, as well as patients who initially reported to the OPD but were subsequently admitted for further care.

Sample size

According to a document by the WHO, “How to investigate drug use in health facilities,” at least 600 prescription encounters should be included in a cross-sectional survey to describe the current prescription pattern [10]. Based on this, we have selected 600 patients as the sample size.

Data collection

Patient data meeting inclusion criteria were recorded, including name, age, sex, diagnosis, and ongoing treatment, on prepared case record forms during outpatient visits. A total of 600 cases were collected. Data were compiled and subjected to descriptive statistical analysis using mean and standard deviation in GraphPad Prism version 9 (GraphPad Software, San Diego, CA). The data were collected by the primary author during outpatient visits. Accuracy was ensured through standardized forms, double-checking by supervisors, data validation checks, and regular quality control audits.

Data analysis

This study provides a comprehensive analysis of drug utilization patterns in affective disorder patients at a tertiary care teaching hospital, contributing valuable insights to the field of psychiatric outpatient care.

Descriptive statistics

Mean and standard deviation were used to describe continuous variables, while frequencies and percentages were used for categorical variables and drug utilization analysis was conducted.

DDD/100 bed-days were calculated using the following equation [11]:

DDD/100 bed days = Total dose in mg during study period × 100 ÷ DDD of drug × Study duration (days) × Bed strength × Average bed occupancy rate.

The acquired dataset underwent a comprehensive analysis, delving into various facets of drug utilization among patients with affective disorders. The study scrutinized the morbidity patterns associated with affective disorders, providing a nuanced understanding of the prevalent health challenges within this population. Subsequent analyses focused on demographic factors, including age and sex distribution, as well as occupational, literacy, and marital status distributions, shedding light on the diverse characteristics of the patient cohort.

The study also delved into the prescription practices, revealing valuable insights into the average number of psychotropic drugs prescribed per encounter. Notably, the percentage of drugs prescribed from the essential drugs list and the utilization of generic names were meticulously examined, offering a glimpse into the adherence to standardized drug practices. The frequency of drug use in affective disorders was explored, alongside a classification of psychotropic drug use within this specific patient group.

Beyond psychotropic medications, the study extended its analysis to non-psychotropic drugs, elucidating the percentage of prescriptions within this category. Detailed examinations of prescribing patterns for psychotropic drugs in affective disorders uncovered key trends, providing a deeper understanding of the therapeutic strategies employed by clinicians.

In addition, the distribution of encounters based on patient and relative counseling was explored, highlighting the role of communication in health care. The study also assessed the average consultation time per encounter, acknowledging the importance of effective physician-patient interactions. Patient confidentiality was maintained by anonymization. Patient identifiers such as names and unique identification numbers were replaced with study-specific codes to anonymize the data. Ethical clearance was provided by the Institutional Ethical Committee on 11/02/2020 with ref no. IEC/Certi/26/01/2020.

## Results

The sociodemographic details of patients with various affective disorders are summarized, as among the total 600 patients surveyed, there were 369 male patients, comprising 61.5% of the total encounters. Female patients were 231 of the total encounters, representing 38.5% of the surveyed population. The majority were married (76.83%), while unmarried patients accounted for 23.17% of the total encounters. In terms of literacy, the majority of patients were found to be literate, constituting 72.3% of the sample, whereas 27.7% were illiterate. In terms of occupation-wise distribution, the majority of patients with various affective disorders were engaged in private employment (40%), followed by housewives (23.33%). Unemployed individuals accounted for 21.17% of the total encounters, while government employees constituted 9.5%. Students made up 6% of the surveyed population.

Among the patients visiting the psychiatric OPD, a comprehensive assessment of morbidity patterns revealed a predominance of bipolar mood disorder (39.83%), followed by depressive disorder (34.67%), mania disorder (17.50%), and schizoaffective disorder (8%).

When considering the distribution of affective disorders by sex, it was observed that bipolar mood disorder exhibited a higher prevalence among male patients (28.83%) compared to female patients (11%). Conversely, depressive disorder demonstrated a higher prevalence among female patients (20.17%) compared to male patients (14.5%). In cases of mania disorder, male patients were notably more prevalent (13.67%) than female patients (3.83%). Similarly, for schizoaffective disorder, male patients (4.5%) outnumbered female patients (3.5%).

A total of 2252 drug prescriptions were analyzed from 600 encounters. The average number of drugs prescribed per encounter was 3.75, with a range of one to seven drugs per encounter. The total cost of drugs amounted to 45735.312 rupees, with an average drug cost per encounter of 76.22 rupees. The total consultation time was 14362 minutes and the average consultation time per encounter was 23.93 minutes.

In our study, a significant proportion of prescribed drugs were sourced from essential drug lists. Specifically, 52.17% of drug types were prescribed from the WHO essential drug list [12], 42.82% from the National List of Essential Medicines of India, and 95.65% from the Gujarat Essential Drug List. Notably, 47.82% of drug types were not prescribed from the WHO essential drug list, 52.17% were not from the National List of Essential Medicines of India, and only 4.34% were not from the Gujarat Essential Drug List.

The most frequently prescribed drug in our study was tablet olanzapine (10.79%), followed by tablet clonazepam (9.1%) and tablet sodium valproate (8.70%). Psychotropic drugs were primarily classified into antipsychotics (35.14%), benzodiazepines (23.39%), antidepressants (21.12%), and mood stabilizers (20.35%).

According to Table 1, out of a total of 208 depressive mood disorder patients, 109 (52.4%) were prescribed dual therapy, followed by monotherapy in 56 (26.92%) encounters, triple therapy in 40 (19.23%) encounters, and one (33.3%) patient was prescribed quadruple therapy.

**Table 1:** Prescribing psychotropic drug pattern in depressive disorder (n = 208)

Category	Drugs	No. of prescriptions
Mono drug therapy	Escitalopram	24 (42.85%)
	Fluoxetine	24 (42.85%)
	Sertraline	8 (14.28%)
Dual drug therapy	Escitalopram + Imipramine	20 (18.348%)
	Fluoxetine + Imipramine	13 (11.926%)
	Escitalopram + Mirtazapine	10 (9.174%)
	Escitalopram + Olanzapine	9 (8.256%)
	Sertraline + Olanzapine	7 (6.422%)
	Sertraline + Imipramine	7 (6.422%)
	Fluoxetine + Mirtazapine	6 (5.504%)
	Fluoxetine + Olanzapine	5 (4.587%)
	Sertraline + Propranolol	5 (4.587%)
	Fluoxetine + Propranolol	5 (4.587%)
	Sertraline + Mirtazapine	5 (4.587%)
	Sertraline + Escitalopram	4 (3.669%)
	Others	13 (11.926%)
Triple drug therapy	Escitalopram + Imipramine + Propranolol	6 (15%)
	Escitalopram + Sertraline + Mirtazapine	4 (10%)
	Fluoxetine + Imipramine + Propranolol	4 (10%)
	Escitalopram + Imipramine + Olanzapine	4 (10%)
	Sertraline + Imipramine + Olanzapine	3 (7.5%)
	Fluoxetine + Escitalopram + Olanzapine	3 (7.5%)
	Sertraline + Imipramine + Propranolol	2 (5%)
	Others	14 (35%)
Quadruple drug therapy	Escitalopram + Risperidone + Olanzapine + Propranolol	1 (33.3%)

Table 2 provides the prescribed daily dose (PDD) of the most frequently prescribed psychotropic drugs for affective disorders. PDD represents the recommended dosage of each drug per day in mg/mmol. Understanding the PDD is crucial for ensuring appropriate dosing and optimizing treatment efficacy while minimizing potential side effects. The PDD varies significantly among different drugs, ranging from small doses like 0.24 mg for alprazolam to larger doses like 1028.06 mg for sodium valproate.

**Table 2:** Prescribed daily dose (PDD), defined daily dose (DDD), and PDD/DDD ratio of psychotropic drugs

Drugs	PDD (mg/mmol)	DDD (mg/mmol)	PDD/DDD ratio
Olanzapine	14.27	10	1.427
Clonazepam	0.50	8	0.062
Sodium valproate	1028.06	1500	0.685
Lorazepam	1.92	2.5	0.768
Escitalopram	19.13	10	1.913
Risperidone	3.144	5	0.628
Haloperidol	8.626	8	1.078
Fluoxetine	48.51	20	2.42
Lithium carbonate	10.35	24	0.431
Carbamazepine	945.65	1000	0.945
Mirtazapine	13.5	30	0.45
Alprazolam	0.24	1	0.24
Sertraline	51.41	50	1.02
Imipramine	39.92	100	0.399
Quetiapine	76.03	400	0.19
Propranolol	51.17	160	0.319
Trihexyphenidyl Hcl (Pacitane)	2.54	10	0.254
Aripiprazole	9.77	15	0.651
Lamotrigine	109.09	300	0.36
Clozapine	160	300	0.53
Amisulpride	100	400	0.25

**Figure 1:**
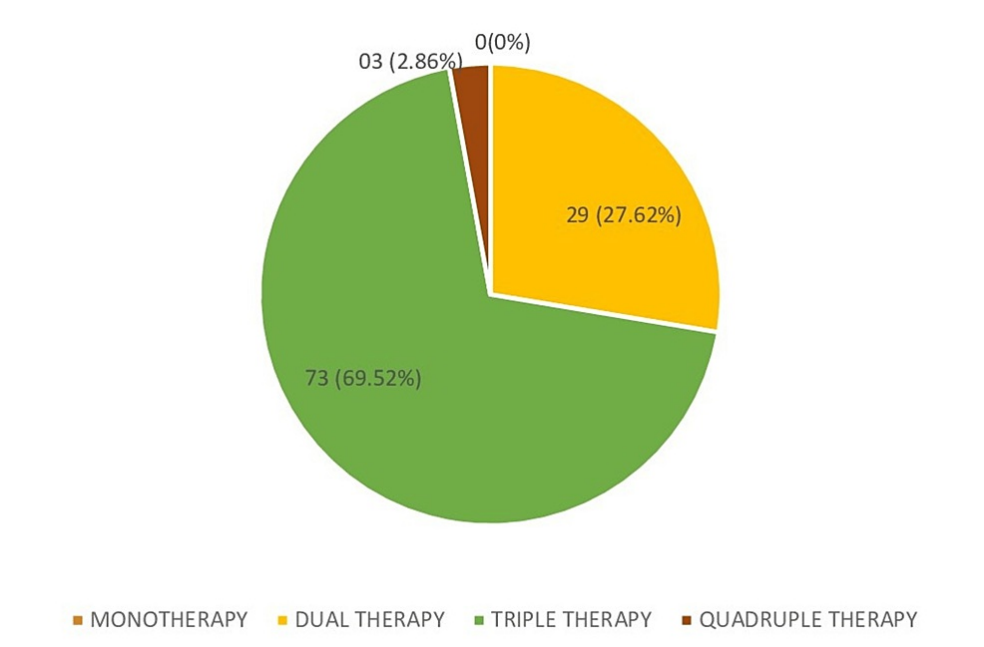
Prescribing psychotropic drug pattern in mania disorder Monotherapy prescribed - 0%.

Among the affective disorder patients, triple therapy was the most common prescription regimen, accounting for 51.33% of encounters, followed by dual therapy (34%), monotherapy (9.34%), and quadruple therapy (5.33%). Specifically, in bipolar mood disorder, triple therapy was the predominant prescription regimen (68.62%), whereas in depressive mood disorder, dual therapy was the most common (52.4%). For mania disorder, as shown in Figure 1, out of a total of 105 mania disorder patients, 73 (69.52%) were prescribed triple therapy, followed by dual therapy in 29 (27.62%) encounters, quadruple therapy in three (2.86%) encounters, and none of them were prescribed monotherapy. In schizoaffective disorder, triple therapy was the most frequent (64.58%).

According to Table 3, 594 (99%) patients or their relatives were counseled regarding the prescribed medications before treatment initiation, 352 (58.67%) were counseled regarding the nature of the disease, and 261 (43.5%) were counseled about lifestyle modifications.

**Table 3:** Distribution according to patient/relative counseling

Patient/relative counseling	Yes	No	Total number of patients
About disease	352 (58.67%)	248 (41.33%)	600 (100%)
About prescription	594 (99%)	6 (1%)	600 (100%)
About lifestyle modification	261 (43.5%)	339 (56.5%)	600 (100%)

**Figure 2:**
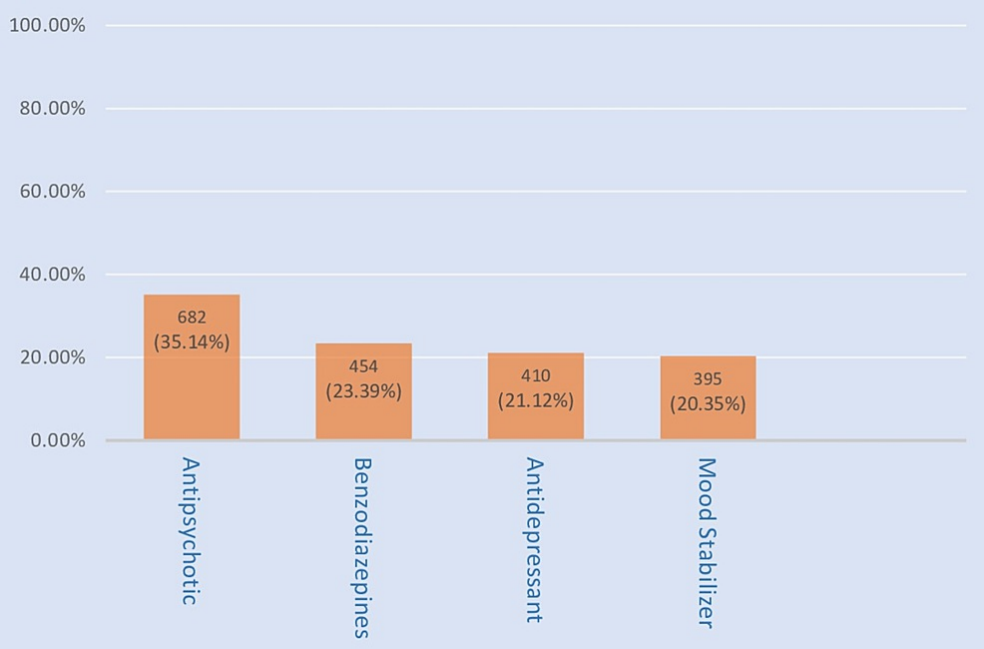
Class of psychotropic drug use in affective disorder

According to Figure 2, which describes the class of psychotropic drugs used in affective disorders, antipsychotics were the most common group prescribed, comprising 682 (35.14%) drugs, followed by benzodiazepines (454, 23.39%) and antidepressants (410, 21.12%) respectively, and mood stabilizers with 395 (20.35%) drugs being the least common group prescribed.

## Discussion

Our study delved into the demographic, diagnostic, and therapeutic aspects of affective disorder patients visiting the psychiatry OPD of a tertiary care teaching hospital. Out of a total of 600 patients, the most frequent morbidity found was bipolar mood disorders, with 239 (39.83%) patients, of which 173 (28.83%) were male whereas 66 (11%) were female, and it was more profound in the age group of 41-50 years (26.36%). In a study carried out by Gawali et al. [13], out of a total of 390 patients, 44 (11.28%) had bipolar mood disorder, of which 31 (7.95%) were males and 13 (3.33%) were females. The disparity in findings between our study and Gawali et al.'s study regarding the prevalence of bipolar mood disorder may be due to differences in sample size and demographic characteristics of the study population.

Among the 600 patients analyzed, the predominant morbidity pattern was observed in bipolar mood disorder (39.83%), followed by depressive disorder (34.67%), mania disorder (17.50%), and schizoaffective disorder (8%). Comparing our findings with existing literature, the prevalence rates were comparable to the study by Jasmine et al. [14], where bipolar disorder accounted for 50.6%, depression for 25.4%, mania for 17.4%, and schizoaffective disorder for 6.6% of enrolled patients. However, Goyal et al. [15] reported lower rates of bipolar disorder (15.19%) and higher rates of depression (27.53%) in contrast to our study. In terms of prescription practices, our study recorded an average of 3.75 drugs per encounter, aligning with similar studies by Jena et al. [16] (3.17 drugs per encounter) and Hussain et al. [17] (2.7 drugs per encounter). Notably, triple psychotropic drug therapy was the most prevalent (51.33%) in our study population, followed by dual therapy (34%), monotherapy (9.34%), and quadruple therapy (5.33%). This could be attributed to the necessity of addressing multiple symptoms simultaneously and achieving acute stability, although it contrasts with findings from Jasmine et al. [14], where monotherapy (56%) was predominant. The disparity in findings regarding the prevalence of multiple drug therapy in our study compared to Jasmine et al.'s study could be attributed to differences in the severity of illness among the study populations, variations in treatment guidelines followed, and the preference of psychiatrists for combination therapy to address complex and refractory symptoms effectively. Additionally, differences in healthcare settings and patient demographics may also contribute to these discrepancies.

Analyzing the PDD against the DDD sheds light on actual drug utilization patterns. For instance, risperidone's PDD of 3.144 mg, compared to a DDD of 5 mg, suggests potential underutilization, indicating the need for reconsideration of prescribing practices. Additionally, counseling sessions played a pivotal role in enhancing patient compliance, with 99% of patients and their relatives receiving pre-counseling sessions regarding prescribed medications, disease nature, and necessary lifestyle modifications. Such interventions are vital for fostering patient understanding and adherence to treatment regimens.

Limitations

This study includes its cross-sectional design, which may limit the ability to establish causality or long-term treatment trends. Additionally, the study's focus on a single tertiary care hospital may restrict the generalizability of findings to other settings or populations. Moreover, the reliance on self-reported data and retrospective analysis could introduce recall bias and inaccuracies in medication history.

Overall, our study underscores the importance of tailored treatment approaches in affective disorders, taking into account demographic variations, prescription practices, and patient education to optimize therapeutic outcomes and ensure patient well-being.

## Conclusions

Our study elucidates the demographic distribution, prescription patterns, and therapeutic approaches in affective disorder patients attending a tertiary care teaching hospital's psychiatry OPD. With bipolar mood disorder emerging as the most prevalent condition, followed by depressive disorder, mania disorder, and schizoaffective disorder, our findings align with existing literature, albeit with some variations. The prescription practices revealed a tendency toward triple psychotropic drug therapy, highlighting the complexity of managing affective disorders. Analysis of PDD against DDD underscored the need for vigilant monitoring and optimization of drug utilization patterns. Furthermore, our study emphasizes the pivotal role of patient counseling in enhancing compliance and treatment outcomes. Overall, our findings contribute to a deeper understanding of affective disorders' management, emphasizing the importance of tailored treatment approaches and patient education for better clinical outcomes.
